# Supplementary material for: MYCN Amplification Is Associated with Reduced Expression of Genes Encoding γ-Secretase Complex and NOTCH Signaling Components in Neuroblastoma
Source: Int J Mol Sci. 2023 May 2;24(9):8141. doi: 10.3390/ijms24098141 (PMC10179553; doi:10.3390/ijms24098141)
Supplement: Supplementary file 1 [file ijms-24-08141-s001.zip › ijms-2210621_R1_Supplementary data file.docx]

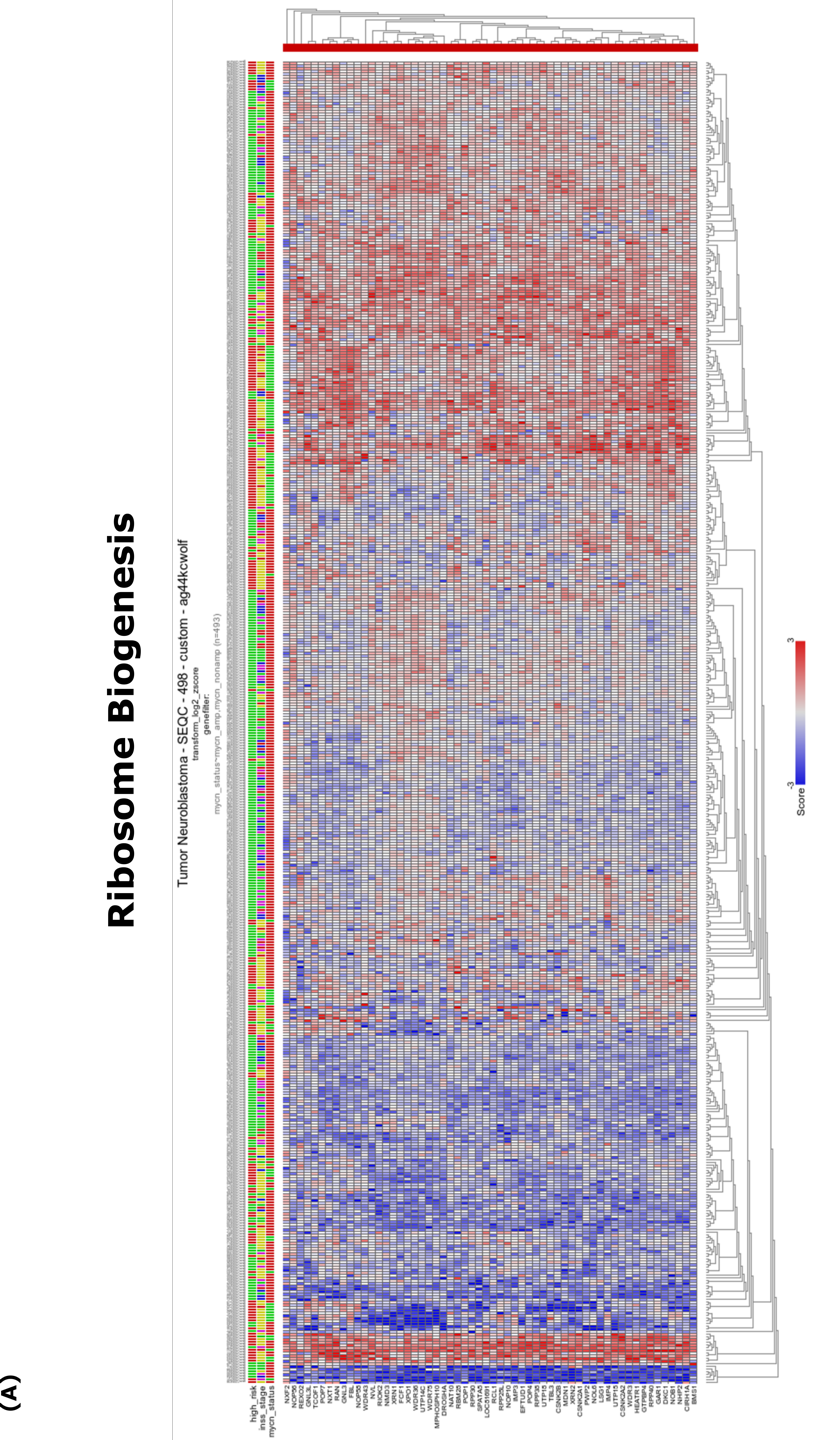


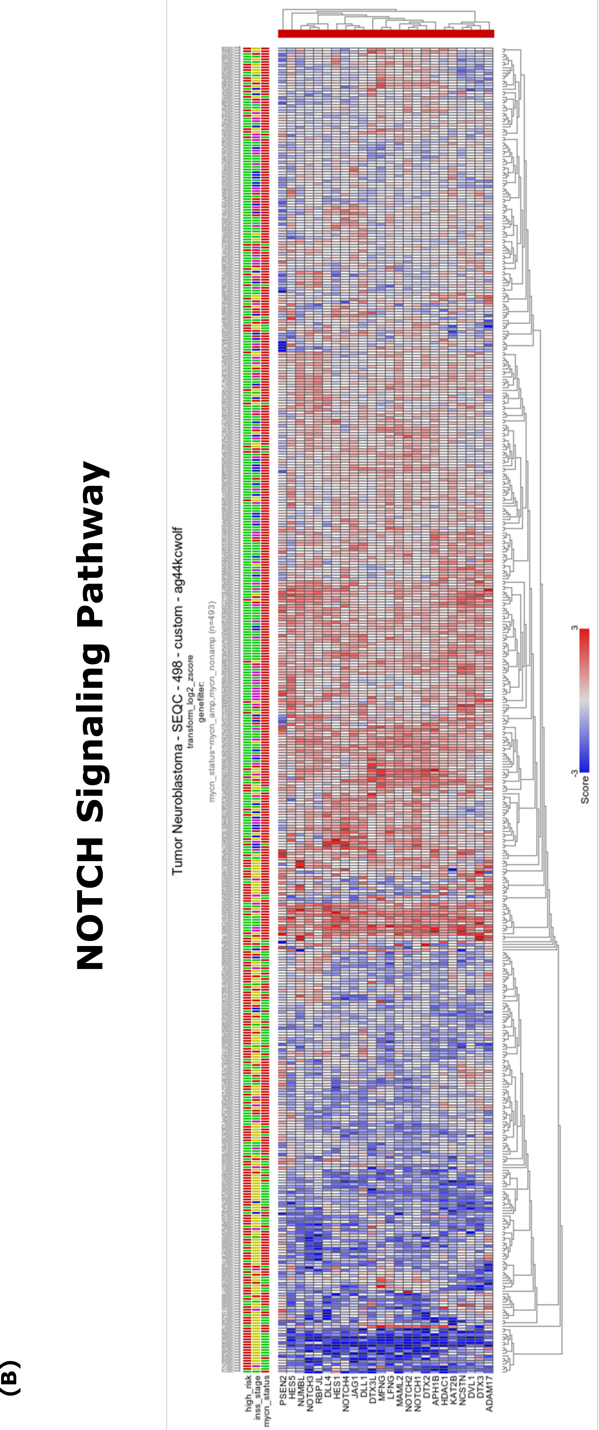


**Figure S1:** Analysis of differentially expressed genes in primary NB tumors in SEQC (GSE49710) dataset reveals increased expression of genes involved in Ribosomal biogenesis (A) and decreased expression of genes involved in Notch signaling pathway (B) in MYCN-amplified tumors (Green labels) when compared to MYCN-non-amplified tumors (Red labels).


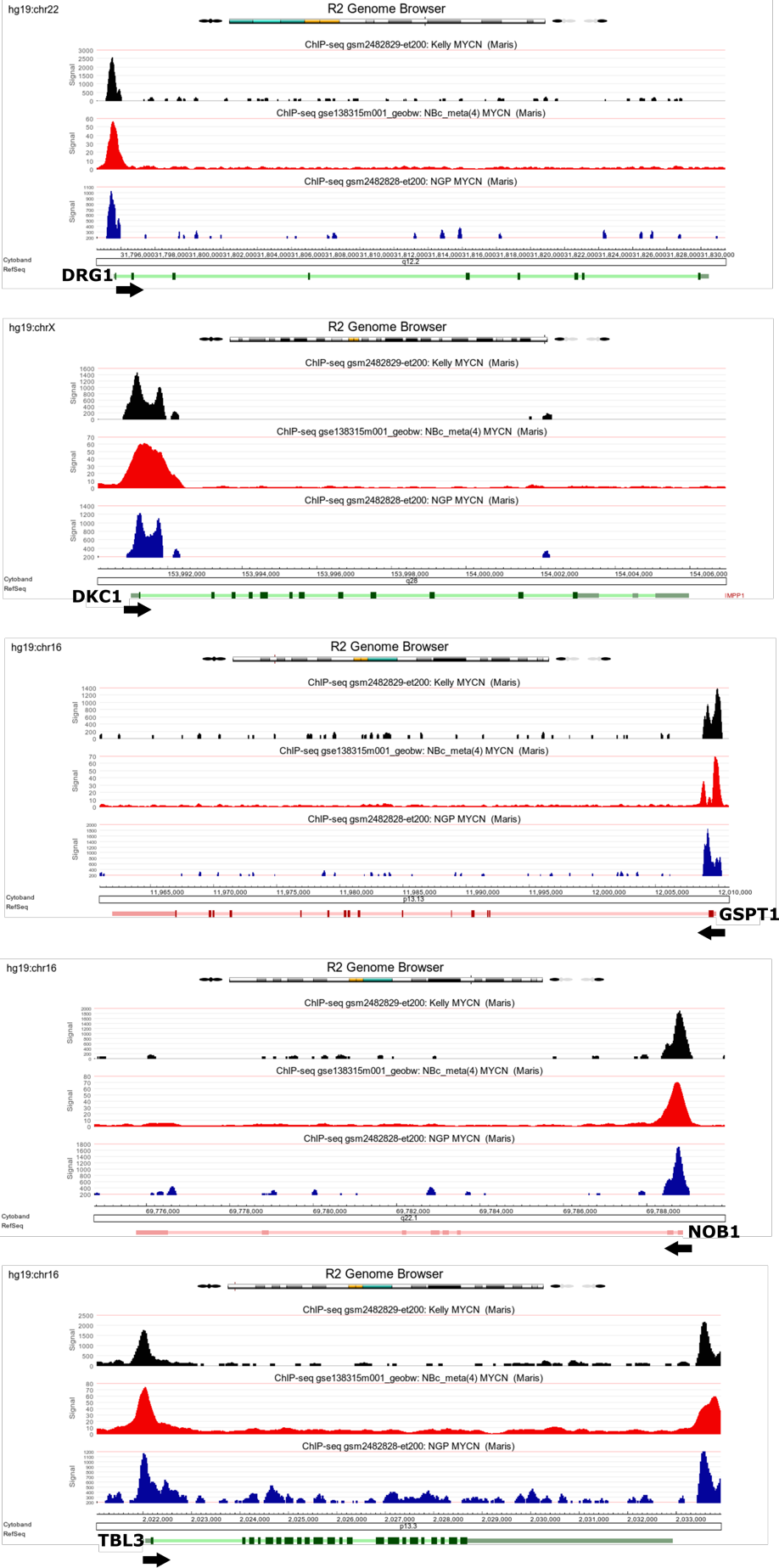


**Figure S2.** R2 genome browser analysis of MYCN ChIP-Seq peaks around the transcription start sites (TSS) of some selected up-regulated gene involved in ribosome biogenesis identified in this study. Arrowhead indicates the direction of transcription.


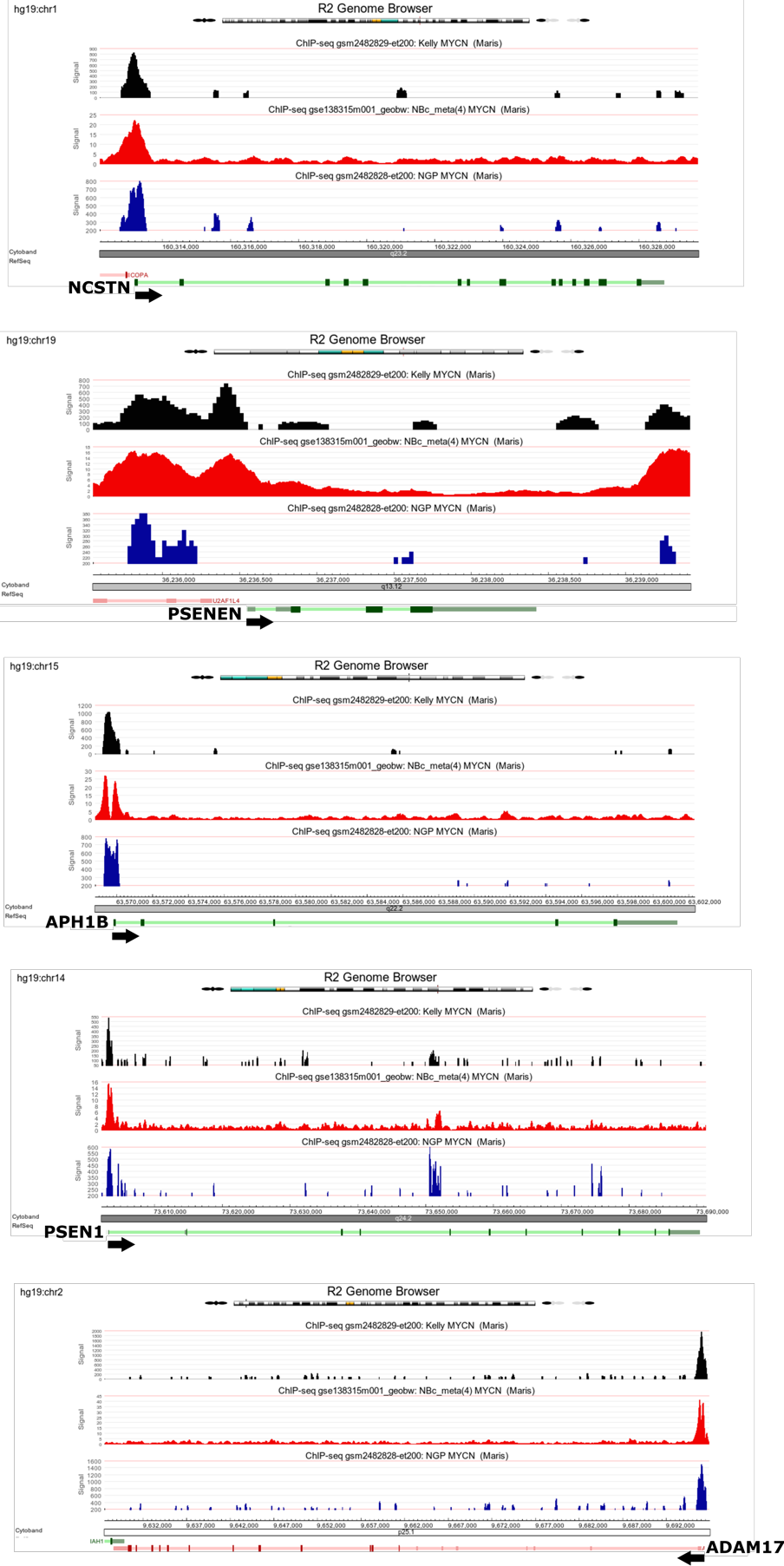


**Figure S3.** R2 genome browser analysis of MYCN ChIP-Seq peaks around the transcription start sites (TSS) of down-regulated genes encoding the γ-secretase complex and ADMA17. Arrowhead indicates the direction of transcription.


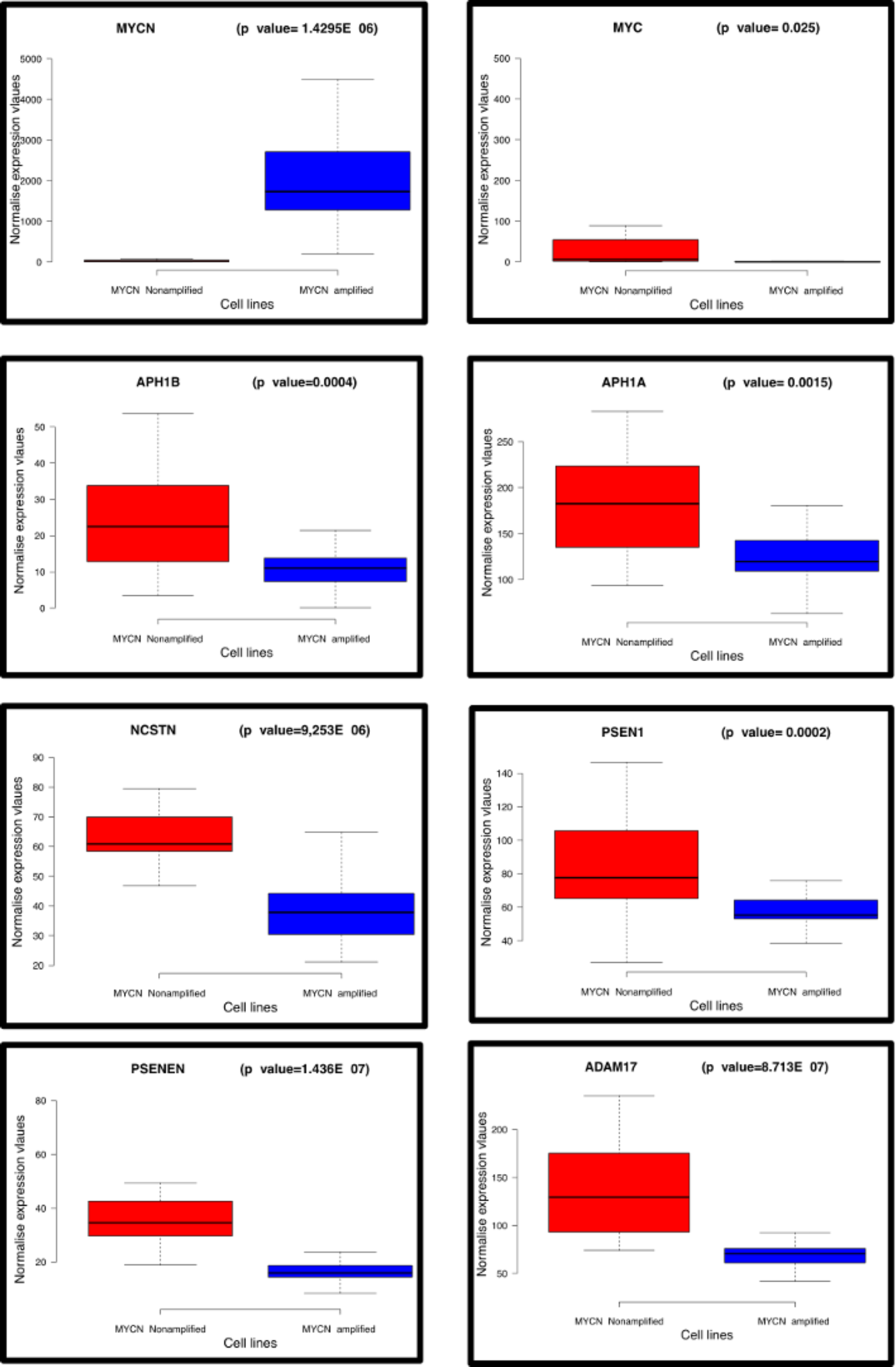


**Figure S4.** Analysis of mRNA levels of MYCN, MYC, ADAM17 and members of the γ-secretase complex in the 39 human NB cell lines used in this study.


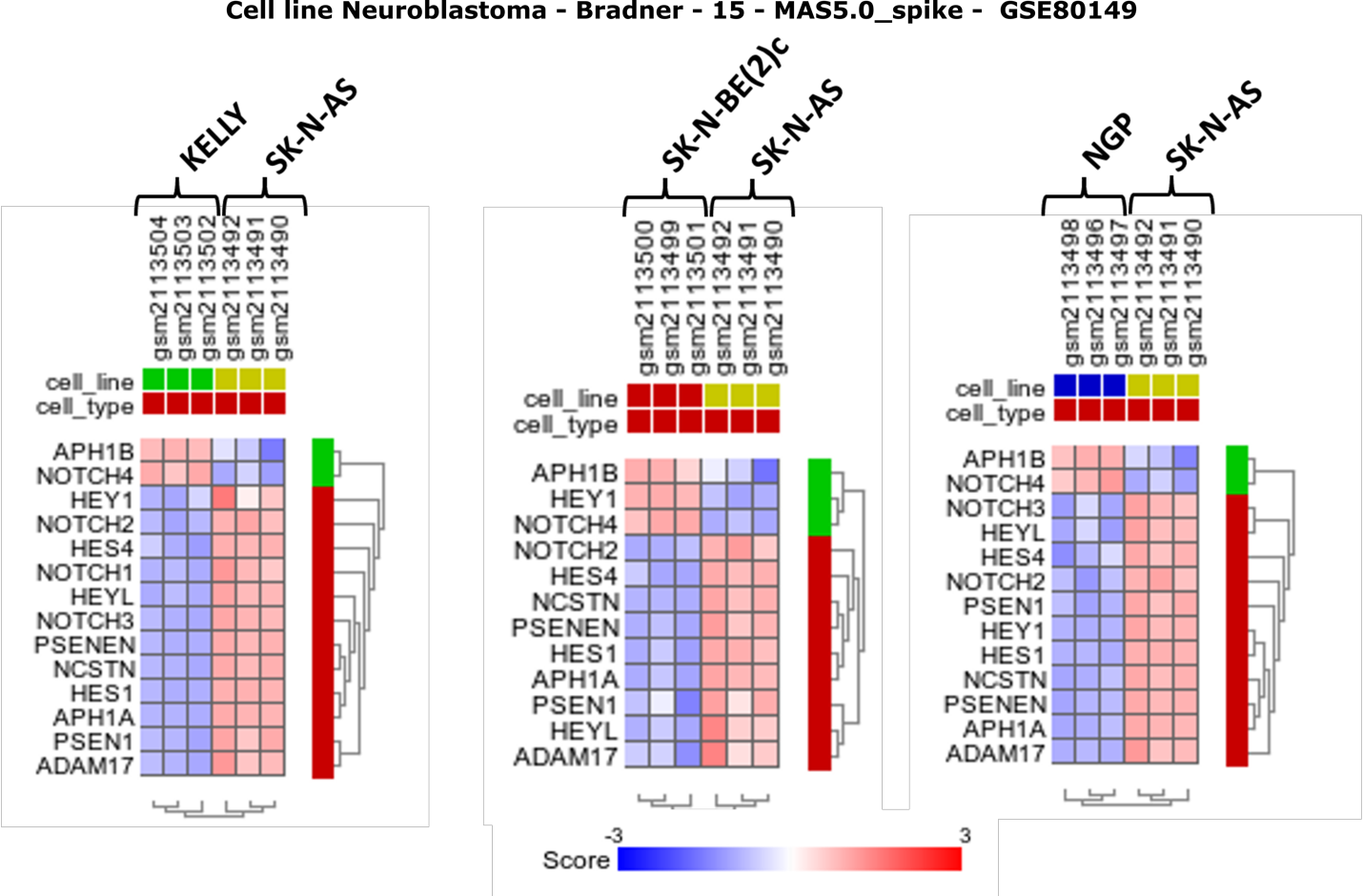


**Figure S5.** MYCN status does not affect global RNA levels and per cell RNA count. Analysis of mRNA levels of several components of the NOTCH signaling pathway in gene expression dataset with spike-in RNA control. KELLY, SK-N-BE(2)c and NGP are MYCN-amplified cell lines, while SK-N-AS is a MYCN-non-amplified cell line.

**
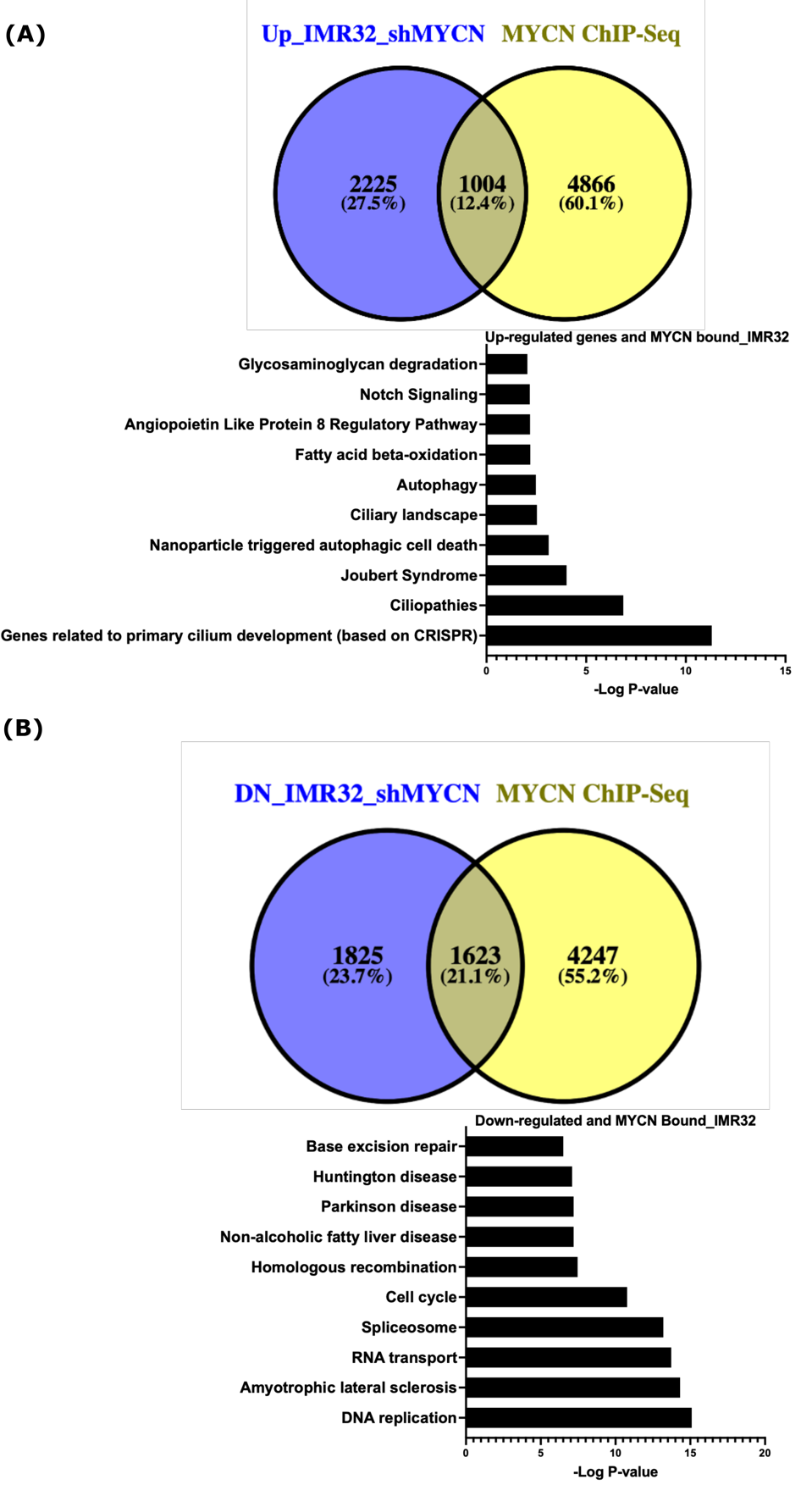
**

**Figure S6.** Analysis of MYCN bound and differentially expressed genes following shRNA-mediated MYCN knockdown in IMR32 cell line. (A) Overlap between our defined MYCN target genes and up-regulated genes in IMR32 in response to MYCN-KD (Top panel). GO analysis of up regulated-MYCN bound genes (Lower panel). (B) Overlap between our defined MYCN target genes and down-regulated genes in IMR32 in response to MYCN-KD (Top panel). GO analysis of down-regulated-MYCN bound genes (Lower panel).


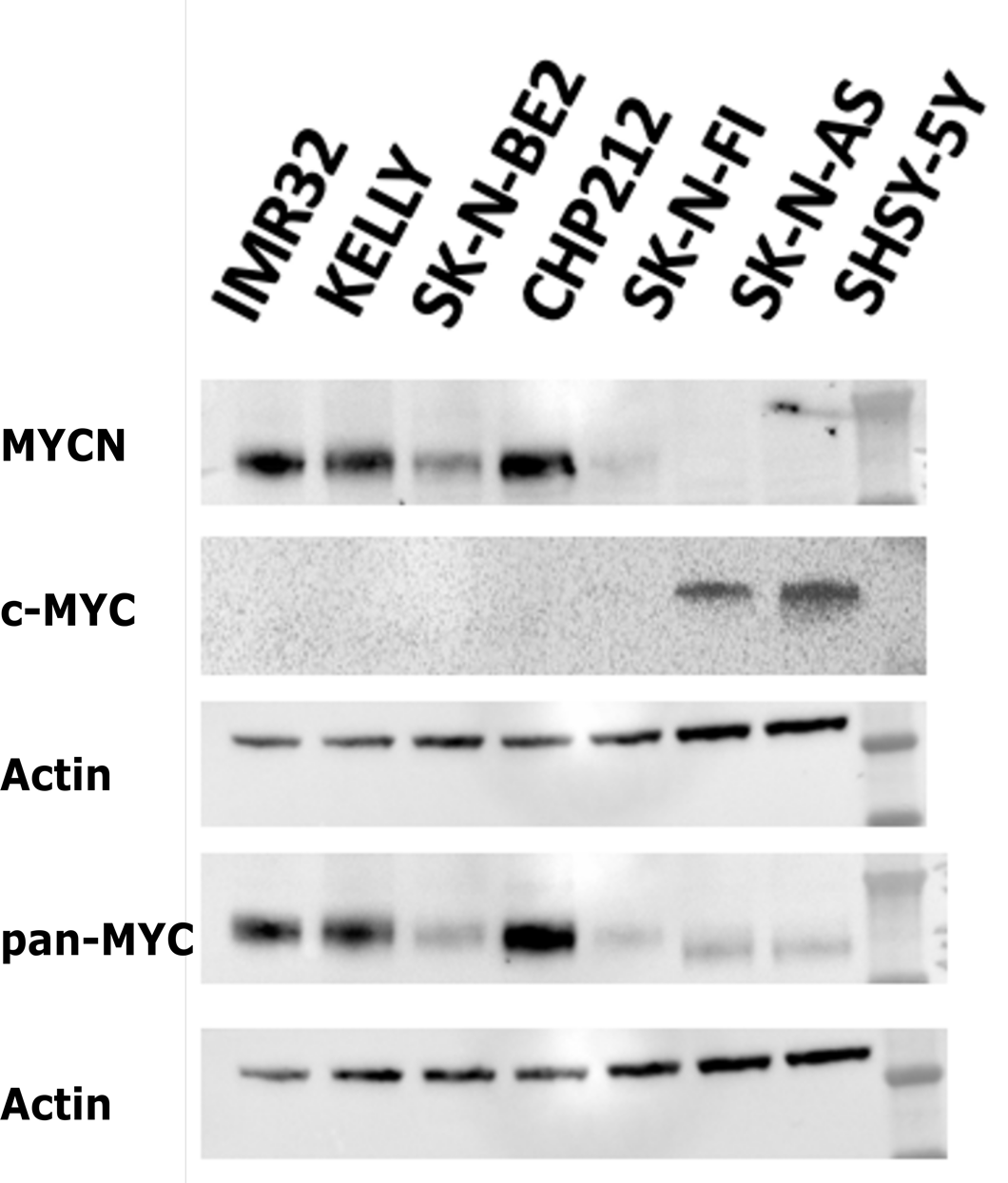


**Figure S7.** Western blot analysis of MYCN, c-MYC, and Pan-MYC protein levels in a set of human NB cell lines used for functional validation in this study.

**
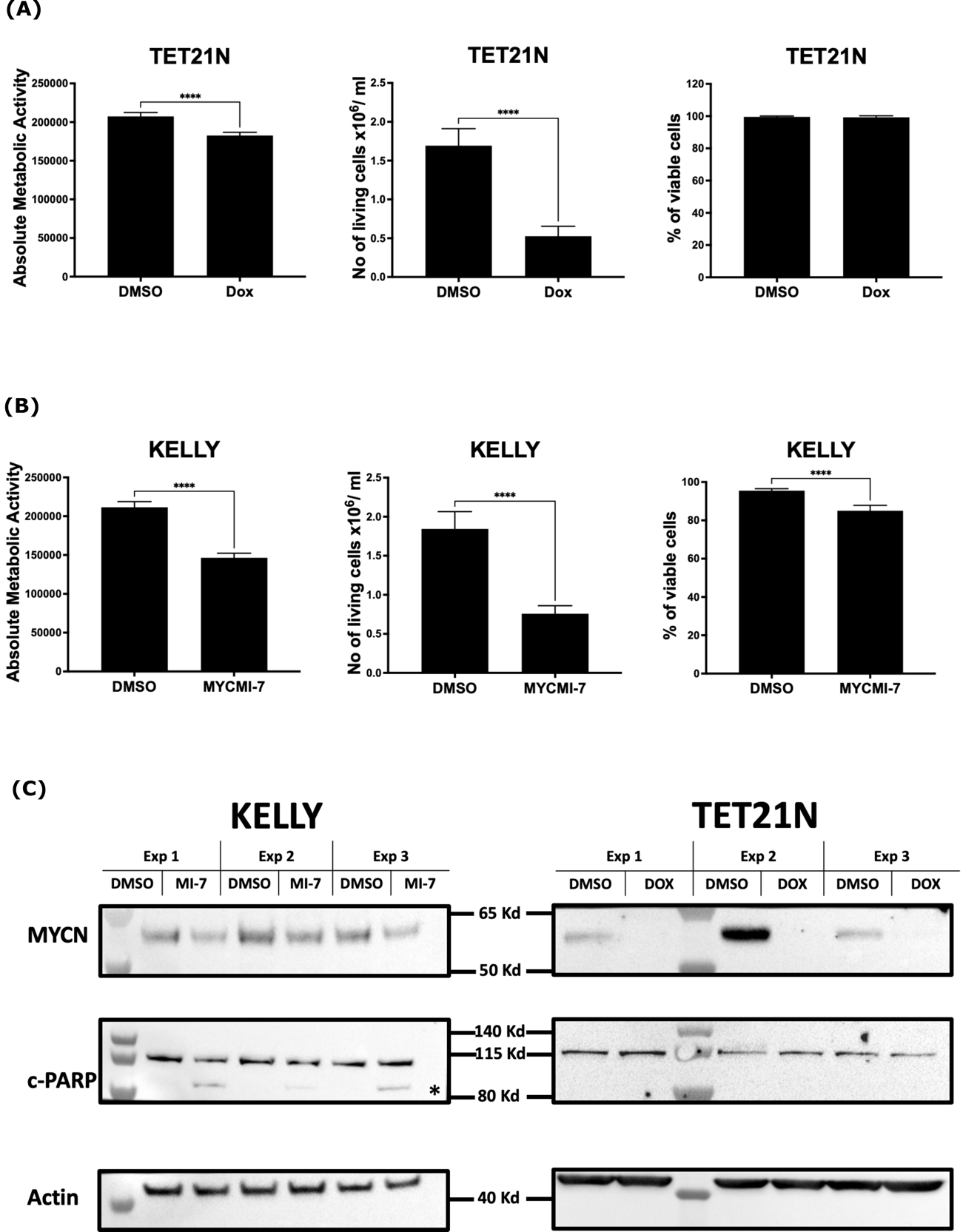
**

**Figure S8.** Impact of MYCN genetic and pharmacological depletion on the growth and survival of NB cell lines. (A)Assessment of cell metabolic activity using resazurin assay, cell proliferation, and viability following MYCN genetic depletion in TET21N. (B) Assessment of cell metabolic activity using resazurin assay, cell proliferation, and viability following MYCN genetic depletion following pharmacological depletion using MYCMY-7 in KELLY. (C) Evaluating cell death by western blot analysis of cleaved PARP in TET21N and KELLY cell lines. **** indicates p-value ≤ 0.0001.

**
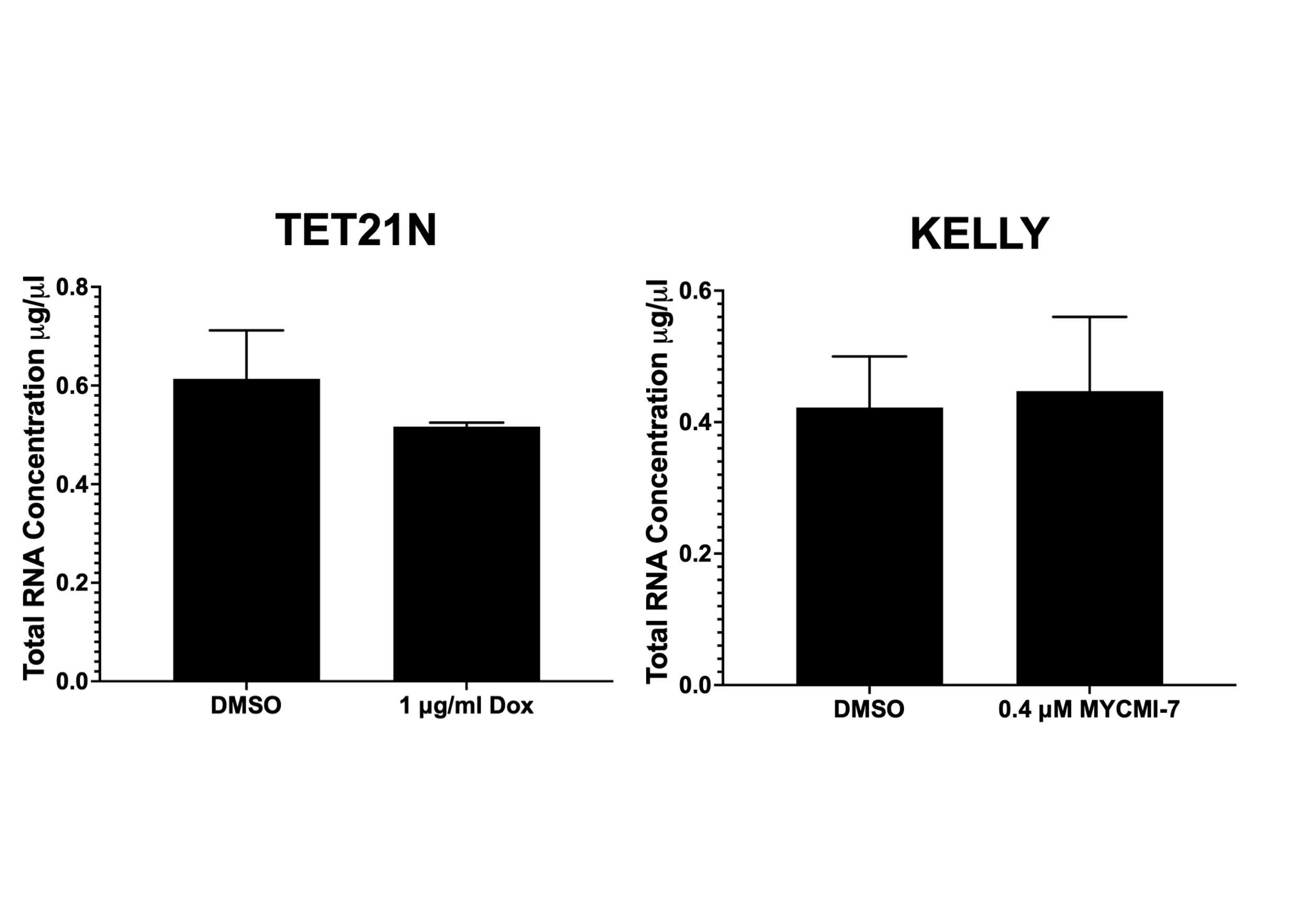
**

**Figure S9.** MYCN status does not affect global RNA levels and per cell RNA count. (A) Total RNA concentration from equal number of cells in TET21N MYCN expressing, with depleted MYCN, following treatment with 1 μg/ml Doxycycline for 72 hours, while KELLY cells were treated with 0.4 μM MYCMI-7 for 72 hours. DMSO was used as control treatment. Error bars represent the standard deviation of three biological experiments.


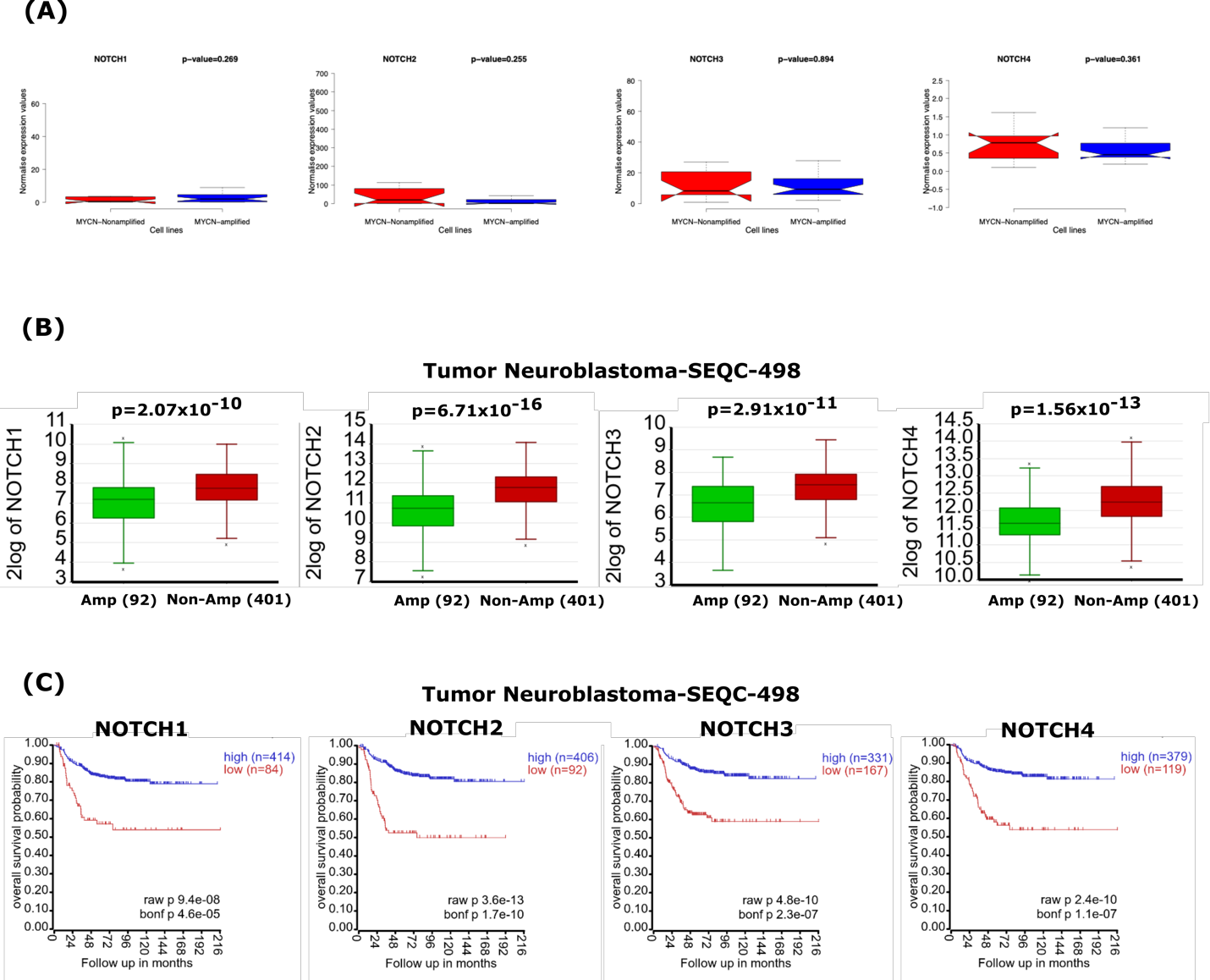


**Figure S10.** NOTCH receptors are lowly expressed in primary neuroblastoma tumors with MYCN-amplification. Analysis of mRNA levels of all NOTCH receptors in the 39 human NB cell line dataset (A), in primary NB tumors with and without MYCN-amplification (B). P-value was calculated using student t-test for the 39 cell line data and one-way anova for the primary tumors data. (C) Kaplan-Meier analysis of overall survival curve from the Tumor Neuroblastoma - SEQC - 498 study based on mRNA expression of all NOTCH receptors. Statistical significance was indicted using Bonferroni Correction of raw P-value.
